# Ets-1 as an early response gene against hypoxia-induced apoptosis in pancreatic *β*-cells

**DOI:** 10.1038/cddis.2015.8

**Published:** 2015-02-19

**Authors:** N Qiao, C Xu, Y-X Zhu, Y Cao, D-C Liu, X Han

**Affiliations:** 1 Key Laboratory of Human Functional Genomics of Jiangsu Province, Jiangsu Diabetes Center, Nanjing Medical University, Nanjing, Jiangsu, China

## Abstract

**Hypoxia complicates islet isolation for transplantation and may contribute to pancreatic *β*-cell failure in type 2 diabetes. Pancreatic *β*-cells are susceptible to hypoxia-induced apoptosis. Severe hypoxic conditions during the immediate post-transplantation period are a main non-immune factor leading to *β*-cell death and islet graft failure. In this study, we identified the transcription factor Ets-1 (v-ets erythroblastosis virus E26 oncogene homolog 1) as an early response gene against hypoxia-induced apoptosis in pancreatic *β*-cells. Hypoxia regulates Ets-1 at multiple levels according to the degree of *β*-cell oxygen deprivation. Moderate hypoxia promotes Ets-1 gene transcription, whereas severe hypoxia promotes its transactivation activity, as well as its ubiquitin-proteasome mediated degradation. This degradation causes a relative insufficiency of Ets-1 activity, and limits the transactivation effect of Ets-1 on downstream hypoxic-inducible genes and its anti-apoptotic function. Overexpression of ectopic Ets-1 in MIN6 and INS-1 cells protects them from severe hypoxia-induced apoptosis in a mitochondria-dependent manner, confirming that a sufficient amount of Ets-1 activity is critical for protection of pancreatic *β*-cells against hypoxic injury. Targeting Ets-1 expression may be a useful strategy for islet graft protection during the immediate post-transplantation period.**

Hypoxia is a common challenge for living organisms that depend on oxygen.^1^ Pancreatic *β*-cells are particularly susceptible to hypoxia owing to their high demand for oxygen to support the mitochondrial respiration and ATP generation required for subsequent insulin secretion.^2, 3^ Moderate hypoxia can be induced in islets in type 2 diabetic models by the increasing demand for insulin secretion,^2, 4^ whereas severe hypoxia occurs in islet grafts during the immediate post-transplantation period ^5, 6, 7^ and leads to *β*-cell apoptosis.^8, 9, 10^

Cells and organisms are able to trigger adaptive responses to help them to cope with hypoxic conditions and cellular reprogramming mediated by transcription factors has a vital role in these responses.^11, 12^ The transcription factor v-ets erythroblastosis virus E26 oncogene homolog 1 (Ets-1) is a member of the Ets family that contains a unique DNA binding domain, the Ets domain.^13^ It is widely expressed in numerous cell types and is involved in a diverse array of biologic functions.^14^ Oikawa *et al.*^15^ first reported that hypoxia induces Ets-1 gene expression via hypoxia-inducible factor 1 (HIF-1) activity. Since then, much research has been focused on the role of Ets-1 in cellular hypoxic responses. Previous studies have indicated that Ets-1 is transcriptionally upregulated and promotes the expression of downstream hypoxia-inducible genes, in both HIF dependent and independent ways.^16, 17^ These Ets-1 target genes participate in a wide range of hypoxic responses, including angiogenesis,^18, 19^ energy metabolism remodeling ^20^ and tumor invasion.^21, 22^

The role of Ets-1 in pancreatic *β*-cells has rarely been studied. In the present study, we reported that hypoxia regulates Ets-1 at multiple levels in pancreatic *β*-cells according to the degree of oxygen deprivation; that is, moderate hypoxia promotes Ets-1 gene transcription, whereas severe hypoxia enhances its transactivation activity. We detected a rapid decrease in the protein level of Ets-1 because of the ubiquitin-proteasome mediated degradation in MIN6 cells or primary cultured islets exposed to severe hypoxia, and all our evidence indicates that this degradation is transcription dependent. The enhanced degradation leads to an insufficiency of Ets-1 protein and limits its transactivation ability and the anti-hypoxic effect. Overexpression of ectopic Ets-1 in MIN6 and INS-1 cells protected them from severe hypoxia-induced apoptosis in a mitochondria-dependent manner, suggesting that Ets-1 insufficiency is a defect in the adaptive responses against hypoxia-induced pancreatic *β*-cell apoptosis.

## Results

### Severe hypoxia causes increased Ets-1 gene expression and decreased protein level in MIN6 cells and primary cultured islets

**Figure 1:**
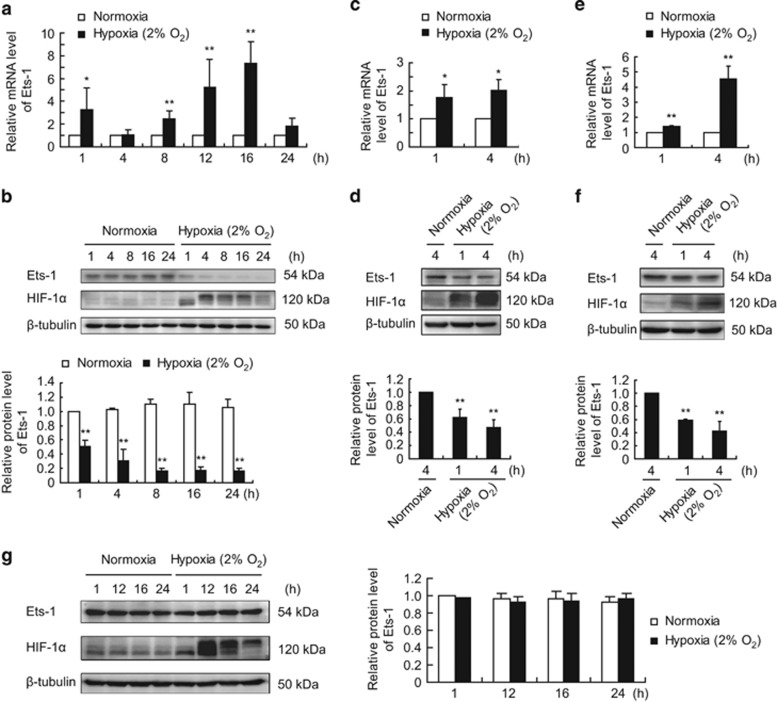
Effects of hypoxia on Ets-1 mRNA and protein levels in MIN6 cells, primary cultured mouse/rat islets and *α*-TC6 cells. MIN6 cells (**a** and **b**), primary cultured mouse (**c** and **d**)/rat (**e** and **f**) islets and *α*-TC6 cells (**g**) were exposed to 2% O_2_ for 1–24 h. (**a**, **c** and **e**) Relative mRNA levels of Ets-1 were quantified by qRT-PCR analysis using *β*-actin as an internal control. The average values and standard deviations (*n*=3) are shown. *and **indicate *P*<0.05 and *P*<0.01, respectively, compared with the normoxic group. (**b**, **d**, **f** and **g**) Total proteins were extracted and analyzed by western blotting analysis. The upper panels (**b**, **d** and **f**) and left panel (**g**) show representative western blots. The lower panels (**b**, **d** and **f**) and right panel (**g**) show the relative quantification of normalized Ets-1 level to *β*-tubulin. The average values and standard deviations (*n*=3) are shown. ** indicate *P*<0.01 compared with the normoxic group

The Ets-1 mRNA level in MIN6 cells increased following the exposure to 2% O_2_ (Figure 1a), but a reverse trend was noted in the Ets-1 protein level; that is, it decreased as early as 1 h following the hypoxia exposure and stayed at a very low level for the rest of the experiment (Figure 1b). Primary cultured mouse (Figures 1c and d) and rat (Figures 1e and f) islets showed a similar response, but the pancreatic *α*-cell line—*α*-TC6 (Figure 1g) showed no decrease in the Ets-1 protein level.

### Ubiquitin-proteasome mediated protein degradation contributes to a severe hypoxia-induced decrease in Ets-1 protein level

**Figure 2:**
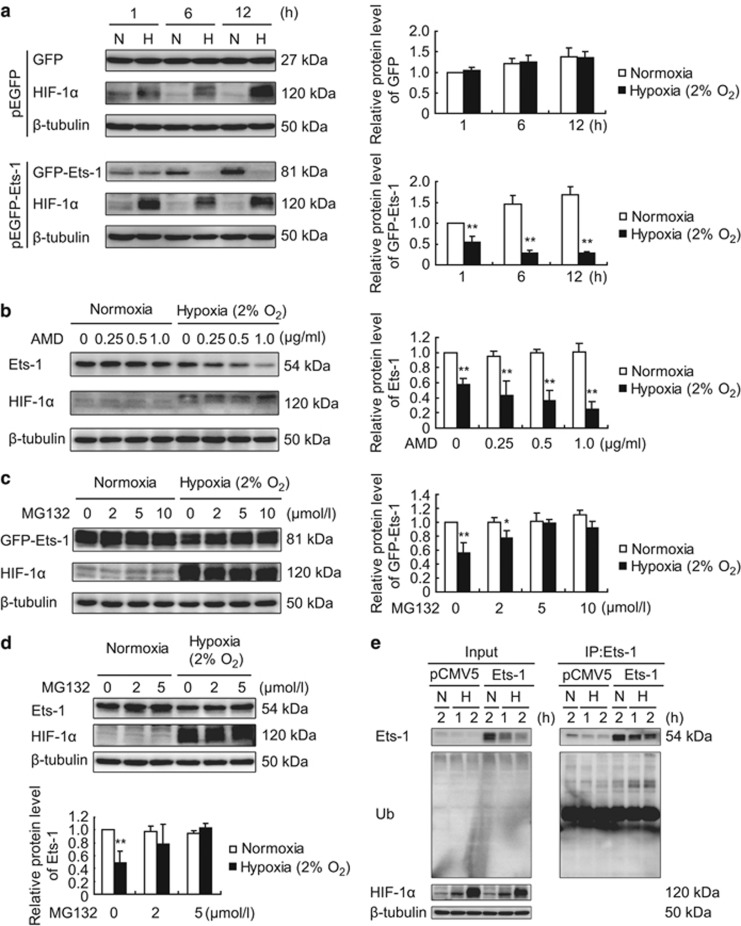
Severe hypoxia promotes ubiquitin-proteasome-mediated degradation of Ets-1 protein in MIN6 cells. AMD, actinomycin D; H, hypoxia; N, normoxia. (**a**) Effect of severe hypoxia on pEGFP vector-mediated ectopic protein expression in MIN6 cells. MIN6 cells were transiently transfected with pEGFP (as a control) or pEGFP-Ets-1. Twenty-four hours after transfection, cells were maintained in normoxic condition or exposed to 2% O_2_ for 1, 6 and 12 h, followed by total protein extraction and western blotting analysis. Anti-GFP antibody was used for immunoblotting of GFP and GFP-Ets-1 fusion protein. (**b**) Actinomycin D enhances hypoxia-induced decrease in Ets-1 protein level. After treatment with actinomycin D at the indicated concentrations, MIN6 cells were immediately exposed to 2% O_2_ for 1 h followed by western blotting analysis. DMSO was used as the solvent control. (**c**–**e**) Ubiquitin-proteasome-mediated degradation of Ets-1 protein. (**c**) MIN6 cells were transiently transfected with pEGFP (as a control) or pEGFP-Ets-1. At 24 h following transfection, the cells were treated with MG132 at the indicated concentrations and then immediately exposed to 2% O_2_ for 1 h. (**d**) MIN6 cells were treated with MG132 at the indicated concentrations and then immediately exposed to 2% O_2_ for 1 h. (**c** and **d**) Total proteins were extracted and the protein levels of GFP-Ets-1 and endogenous Ets-1 were analyzed by western blotting using anti-Ets-1 antibody. (**a**–**d**) The left panels (**a**–**c**) and upper panel (**d**) show representative western blots. The right panels (**a**–**c**) and lower panel (**d**) show the relative quantification of normalized GFP, GFP-Ets-1 and Ets-1 levels to *β*-tubulin. The average values and standard deviations (*n*=3) are shown. *and **indicate *P*<0.05 and *P*<0.01, respectively, compared with the normoxic group. (**e**) MIN6 cells were transiently transfected with pCMV5 (as a control) or pCMV5-Ets-1. At 24 h following transfection, the cells were exposed to 2% O_2_ for 1 h or 2 h, followed by immunoprecipitation. Ets-1 proteins were immunoprecipitated using anti-Ets-1 antibody and then analyzed by western blotting using an anti-ubiquitin antibody to determine the degree of protein polyubiquitination

When we used pEGFP as the expression vector and ectopically expressed GPF or the GFP-Ets-1 fusion protein in MIN6 cells, we found that the expression profile of the GFP protein did not change following a 12 h exposure to 2% O_2_ (Figure 2a). This finding confirmed that the gene expression controlled by the CMV (Cytomegalovirus) promoter would not be influenced by our experimental conditions. By contrast, we saw a rapid and persistent decrease in GFP-Ets-1 protein level (Figure 2a), suggesting enhanced Ets-1 protein degradation. Furthermore, the transcription inhibitor—actinomycin D—dose-dependently enhanced this hypoxia-induced decrease in Ets-1 protein level (Figure 2b).

We then investigated the potential degradation pathway for Ets-1 protein following hypoxia. Addition of MG132 (Carbobenzoxy-Leu-Leu-leucinal), used as a proteasome inhibitor, dose-dependently reversed the severe hypoxia-induced ectopic GFP-Ets-1 protein degradation in MIN6 cells (Figure 2c), suggesting that the Ets-1 protein was degraded via the ubiquitin-proteasome pathway. Similar result was obtained on endogenous Ets-1 protein (Figure 2d). We also detected an accumulation of the polyubiquitinated form of the Ets-1 protein in MIN6 cells following severe hypoxia (Figure 2e). This observation indicated that hypoxia promoted Ets-1 protein ubiquitination and subsequent degradation by the proteasome in MIN6 cells.

### Severe hypoxia enhances the transactivation activity of Ets-1 in MIN6 cells

**Figure 3:**
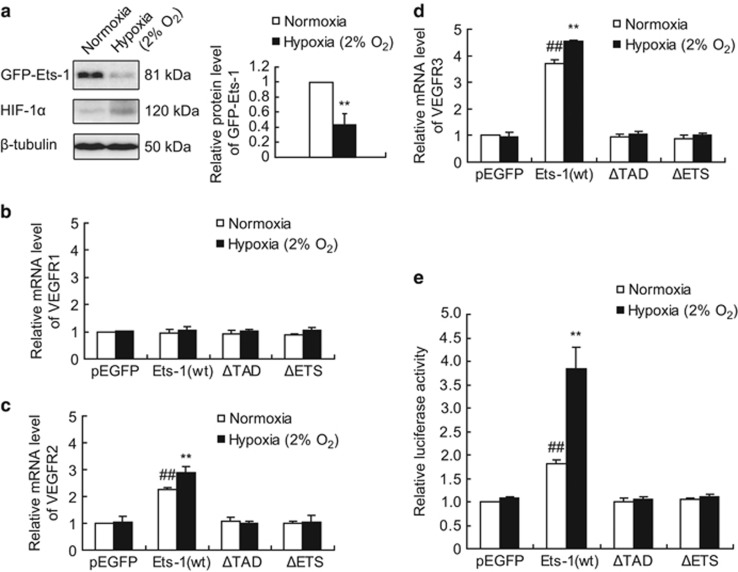
Regulatory effect of Ets-1 on VEGFRs gene transcription and VEGFR3 luciferase reporter activity. (**a**) Relative quantification of GFP-Ets-1 protein. MIN6 cells were transfected with pEGFP-ETS-1^WT^. At 24 h following transfection, cells were maintained in normoxic condition or exposed to 2% O_2_ for 1 h, followed by western blotting analysis. The left panel shows a representative western blot. The right panel shows the relative quantification of normalized GFP-Ets-1 level to *β*-tubulin. The average values and standard deviations (*n*=3) are shown. **indicates *P*<0.01 compared with the normoxic group. (**b**–**d**) MIN6 cells were transiently transfected with pEGFP (as a control), pEGFP-Ets-1^WT^, pEGFP-ETS-1^ΔTAD^ and pEGFP-ETS-1^ΔETS^. At 24 h following transfection, cells were maintained in normoxic condition or exposed to 2% O_2_ for 1 h, followed by total RNA extraction and qRT-PCR analysis. Relative mRNA levels of (**b**) VEGFR1, (**c**) VEGFR2 and (**d**) VEGFR3 were compared between groups using *β*-actin as an internal control. The average values and standard deviations (*n*=3) are shown. **indicates *P*<0.01 compared with the normoxic group. ^##^indicates *P*<0.01 compared with the pEGFP group. (**e**) MIN6 cells were co-transfected with VEGFR3-Luc, pEGFP/pEGFP-Ets-1^WT^/pEGFP-ETS-1^ΔTAD^/pEGFP-ETS-1^ΔETS^ and a *β*-galactosidase expressing plasmid. At 24 h following transfection, cells were maintained in normoxic condition or exposed to 2% O_2_ for 1 h, followed by luciferase reporter assay. The average values and standard deviations (*n*=3) are shown. **indicates *P*<0.01 compared with the normoxic group. ^##^indicates *P*<0.01 compared with the pEGFP group

We assessed the transactivation effect of Ets-1 on vascular endothelial growth factors (VEGFRs), which are the direct target genes of Ets-1.^18, 23, 24, 25, 26^ As shown in Figures 3b–d, overexpression of wild-type Ets-1 in MIN6 cells caused a marked increase in the mRNA level of VEGFR2 and VEGFR3, but not of VEGFR1. By contrast, overexpression of Ets-1^ΔTAD^ (transactivation domain) and Ets-1^ΔETS^, two deletion mutants of Ets-1 that cause a loss in transactivation activity and DNA binding activity, respectively,^14^ did not show any transactivation effect on the VEGFR2 or VEGFR3 genes, indicating a regulatory effect of Ets-1 on VEGFR gene transcription in MIN6 cells.

When the cells transfected with wild-type Ets-1 were exposed to 2% O_2_ for 1 h and the relative mRNA levels of VEGFRs were compared between the normoxic and hypoxic group, cells subjected to hypoxia showed significantly reduced protein level of ectopic Ets-1 (by 57% Figure 3a), but increased transcription of the VEGFR2 (Figure 3c) and VEGFR3 (Figure 3d) genes, indicating an enhancement of the transactivation activity of Ets-1.

We next performed luciferase reporter assay using a luciferase reporter construction driven by the VEGFR3 promoter, to further confirm the change of the transactivation activity of Ets-1 during hypoxia. As expected, Ets-1^WT^ but not Ets-1^ΔTAD^ and Ets-1^ΔETS^ upregulated VEGFR3 luciferase reporter activity in MIN6 cells (Figure 3e). When we exposed the cells to 2% O_2_ for 1 h, the luciferase reporter activity was further upregulated in Ets-1^WT^ overexpression group (Figure 3e). Together, the quantitative real-time polymerase chain reaction (qRT-PCR) assays and the luciferase reporter assays demonstrated that the transactivation activity of Ets-1 was promoted by hypoxia.

### Hypoxia-induced Ets-1 degradation is transcription dependent

**Figure 4:**
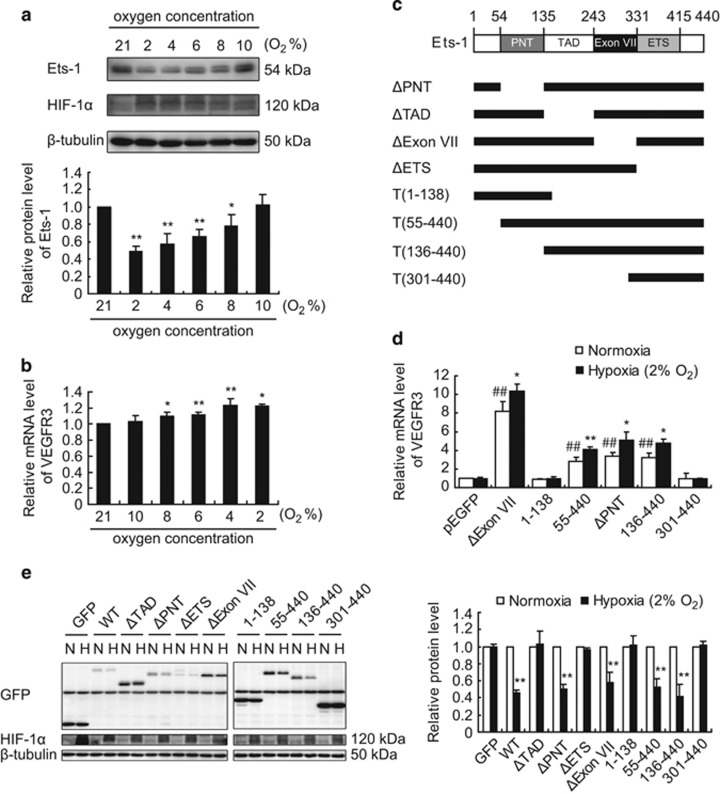
Severe hypoxia-induced degradation of Ets-1 is correlated to its transactivation activity. (**a**) Effect of oxygen concentration on the protein level of Ets-1. MIN6 cells were exposed to 2, 4, 6, 8 and 10% O_2_ for 1 h. Total proteins were extracted and analyzed by western blotting. (**b**) Effect of oxygen concentration on the transactivation activity of Ets-1. MIN6 cells were transfected with pEGFP-Ets-1^WT^. At 24 h following transfection, cells were exposed to 2, 4, 6, 8 and 10% O_2_ for 1 h, followed by RNA extraction and qRT-PCR analysis. The average values and standard deviations (*n*=3) are shown. *and **indicate *P*<0.05 and *P*<0.01, respectively, compared with the normoxic group. (**c**) Schematic diagrams of the Ets-1 constructions used. ETS, ETS domain; PNT, pointed domain; TAD, transactivation domain. (**d** and **e**) Deletion mutagenesis blocking the transactivation activity of Ets-1 stabilized the proteins during severe hypoxia exposure. MIN6 cells were transfected with plasmids as indicated. At 24 h following transfection, MIN6 cells were exposed to 2% O_2_ for 1 h, followed by analysis by (**d**) qRT-PCR and (**e**) western blotting, respectively. (**d**) The average values and standard deviations (*n*=3) are shown. *and **indicate *P*<0.05 and *P*<0.01, respectively, compared with the normoxic group. ^##^indicates *P*<0.01 compared with the pEGFP group. (**a** and **e**) The upper panel (**a**) and left panel (**e**) show a representative western blot. The lower panel (**a**) and right (**e**) panel show the relative quantification of normalized Ets-1, Ets-1 deletion mutants and GFP levels to *β*-tubulin. The average values and standard deviations (*n*=3) are shown. *and **indicate *P*<0.05 and *P*<0.01, respectively, compared with the normoxic group

We investigated whether severe hypoxia-induced degradation of Ets-1 protein is transcription dependent, by first screening for the degree of oxygen deprivation that would lead to activation and degradation of Ets-1. As shown in Figures 4a and b, an oxygen concentration below 10% was required to observe a decrease in the protein level of Ets-1 and an increase in the transactivation activity of it; both responses occurred in an oxygen concentration dependent manner. These results suggested a correlation between the transactivation activity of Ets-1 and its degradation.

We confirmed this correlation in our array of plasmids expressing different deletion mutants of Ets-1 according to its functional domains (Figure 4c),^14, 27^ as we found that only transcriptionally inactive mutants (i.e., Ets-1^ΔTAD^, Ets-1^ΔETS^, Ets-1^1–138^ and Ets-1^301–400^) were resistant to severe hypoxia-induced Ets-1 degradation (Figures 3d, 4d and e). Together, these results indicated a transcription dependency for hypoxia-induced Ets-1 degradation.

### Ets-1 overexpression protects MIN6 cells from severe hypoxia-induced apoptosis in a mitochondria-dependent manner

**Figure 5:**
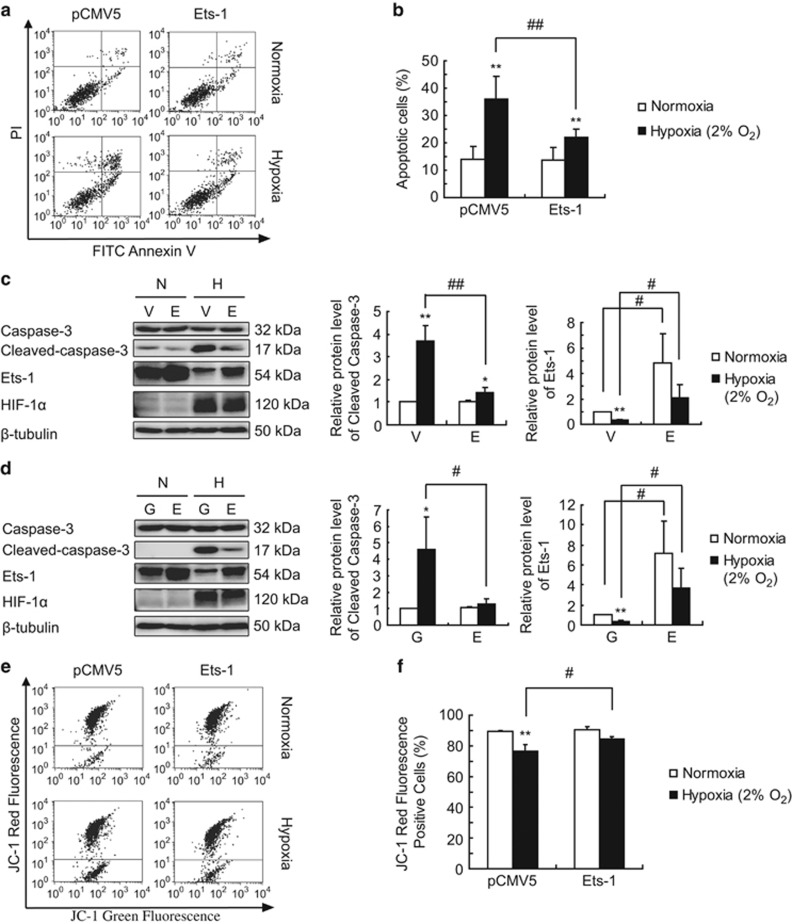
Protective effect of Ets-1 on severe hypoxia-induced pancreatic *β*-cell apoptosis. (**a**) MIN6 cells were transiently transfected with pCMV5 (as a control) and pCMV5-Ets-1. At 24 h following transfection, the cells were exposed to 2% O_2_ for 24 h, followed by annexin V-FITC/PI staining and flow cytometry analysis to determine the percentage of apoptotic cells. Dots in the lower right quadrant indicate annexin V^+^/PI^−^, early apoptotic cells, whereas dots in the upper right quadrant indicate annexin V^+^/PI^+^, late apoptotic cells. (**b**) Statistical graph of apoptotic cells as percentages. The *Y*-axis, (i.e., % apoptotic cells) indicates the percentage of early plus late apoptotic cells. The average values and standard deviations (*n*=3) are shown. **indicates *P*<0.01 compared with the normoxic group. ^##^indicates *P*<0.01 compared with the pCMV5 group. (**c**) Min6 cells were transfected with pCMV5 (as a control) and pCMV5-Ets-1. V=pCMV5; E=pCMV5-Ets-1. (**d**) INS-1 cells were infected with AdV-GFP (as a control) and AdV-Ets-1. G=AdV-GFP; E=AdV-Ets-1. (**c** and **d**) At 24 h following transfection or infection, total proteins were extracted and analyzed by western blotting using the indicated antibodies. The left panels show representative western blot. The right panels show the relative quantification of normalized Cleaved-Capase-3 and Ets-1 levels to *β*-tubulin. The average values and standard deviations (*n*=3) are shown. *and **indicate *P*<0.05 and *P*<0.01, respectively, compared with the normoxic group. ^#^and ^##^indicates *P*<0.05 and *P*<0.01, respectively, compared with the pCMV5 group (**c**) or the AdV-GFP group (**d**). (**e**) MIN6 cells were treated as described in **a**, and then collected and stained with JC-1, followed by flow cytometry analysis. (**f**) Statistical graph of JC-1 red fluorescence positive cells as percentages. The average values and standard deviations (*n*=3) are shown. **indicates *P*<0.01 compared with the normoxic group. ^#^indicates *P*<0.05 compared with the pCMV5 group

Exposure to 2% O_2_ induced apoptosis in MIN6 cells after 12 h (Supplementary Figures 1a–c). When we examined the hypoxia-induced apoptotic phenotypes in MIN6 cells with or without Ets-1 overexpression, we found that the annexin V-FITC/PI (Propidium Iodide) staining (Figures 5a and b) indicated that Ets-1 overexpression partially reversed the apoptosis of MIN6 cells induced by severe hypoxia (represented by ‘annexin V^+^/PI^−^' dots plus ‘annexin V^+^/PI^+^' dots). Consistent with the annexin V-FITC/PI staining results, Ets-1 overexpression in MIN6 cells mediated by the pCMV5 (Figure 5c) and by recombinant adenovirus in INS-1 cells (Figure 5d) partially reversed caspase-3 cleavage induced by severe hypoxia in these cells. These results indicated that a threshold amount of Ets-1 is needed for survival of pancreatic *β*-cells against hypoxia-induced apoptosis.

The JC-1 staining and flow cytometry analysis revealed an obvious disruption of the mitochondrial membrane potential in MIN6 cells exposed to 2% O_2_ for 12 h. Ets-1 overexpression partially reversed the hypoxia-induced mitochondrial membrane potential disruption (Figures 5e and f).

### Hypoxia regulates Ets-1 at multi-levels according to the degree of oxygen deprivation

**Figure 6:**
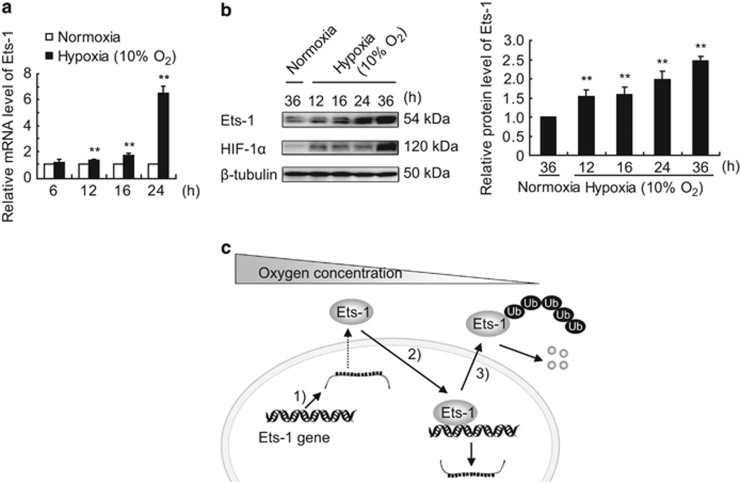
The pattern of multi-level regulation on Ets-1 during hypoxia. (**a**) Effect of moderate hypoxia on Ets-1 gene transcription in MIN6 cells. MIN6 cells were exposed to 10% O_2_ for 6, 12, 16 and 24 h. Relative mRNA levels of Ets-1 were quantified by qRT-PCR analysis. The average values and standard deviations (*n*=3) are shown. **indicates *P*<0.01, compared with the normoxic group. (**b**) Effect of moderate hypoxia on Ets-1 protein level in MIN6 cells. MIN6 cells were exposed to 10% O_2_ for 12, 16, 24 and 36 h, followed by western blotting analysis. The left panel shows a representative western blot. The right panel shows the relative quantification of normalized Ets-1 level to *β*-tubulin. The average values and standard deviations (*n*=3) are shown. **indicate *P*<0.01 compared with the normoxic group. (**c**) A summary of the multi-level regulation of Ets-1 during hypoxia. (1) Moderate hypoxia promotes Ets-1 gene transcription. (2) Severe hypoxia promotes the transcriptional potential of Ets-1 on hypoxic-inducible genes. (3) Severe hypoxia leads to ubiquitin-proteasome mediated degradation of Ets-1 protein

Figure 4a shows that only severe hypoxia (O_2_%<10) led to a rapid decrease (within 1 h) in Ets-1 protein level. In fact, prolonged exposure of MIN6 cells to moderate hypoxia (10% O_2_) resulted in an accumulation of Ets-1 protein (Figure 6b) owing to the enhanced Ets-1 gene transcription (Figure 6a) in MIN6 cells, indicating a multi-level regulation of Ets-1 according to the degree of oxygen deprivation. As shown in Figure 6c, moderate hypoxia (10% O_2_) promoted Ets-1 gene transcription, whereas severe hypoxia (O_2_%<10) resulted in a further enhancement in its transactivation activity. Severe hypoxia also led to Ets-1 protein degradation via the ubiquitin-proteasome pathway.

## Discussion

We demonstrated here that Ets-1 is an early response gene against hypoxia-induced apoptosis in pancreatic *β*-cells. Severe hypoxia promotes the transcription and the transactivation activity of Ets-1 quickly, but also enhances its concomitant transcription-dependent degradation by the ubiquitin-proteasome system. The net effect of these two processes is a relative insufficiency of Ets-1 activity in *β*-cell hypoxic response. Overexpression of Ets-1 reverses hypoxia-induced *β*-cell apoptosis, and confirms the importance of a threshold amount of Ets-1 activity as a cellular defense against apoptosis.

Pancreatic *β*-cells are known to be highly susceptible to hypoxia. Severe hypoxia occurs in islet grafts during the immediate post-transplantation period ^5, 6, 7^ and is a main non-immune factor contributing to islet graft failure.^28, 29^ The present study identified that a relative insufficiency of Ets-1 in *β*-cells during hypoxia can act as a defect in the cellular defense against apoptosis. Ets-1 is strongly induced in many types of cells during hypoxia.^15, 30, 31, 32^ However, contrary to previous studies, we detected a rapid decrease in the protein level of Ets-1 in MIN6 cells and primary cultured mouse/rat islets exposed to severe hypoxia. Moreover, overexpression of Ets-1 in MIN6 and INS-1 cells could protect them from hypoxia-induced apoptosis, confirming the importance of a threshold amount of Ets-1 activity as a cellular defense against apoptosis.

The present study showed that severe hypoxia induced a decrease in Ets-1 protein level but this was not caused by attenuated transcription of Ets-1 gene, but rather by enhanced transcription-dependent degradation of the Ets-1 protein via the ubiquitin-proteasome pathway. Severe hypoxia, in fact, induced a rapid increase in Ets-1 at both the transcription and transactivation activity levels within 1 h. This pattern defines Ets-1 as an early response gene in pancreatic *β*-cell hypoxic responses. Previous studies ignored changes in the transactivation activity of Ets-1 during hypoxia in favor of its obviously enhanced expression. Hypoxia-induced increases in the transactivation activity of Ets-1 may also occur in other cell lines besides pancreatic *β*-cells and this should be studied further.

Many transcription factors, particularly those involved in cell cycle control and stress defense, are unstable proteins targeted by the ubiquitin-proteasome system.^33, 34^ Rapid turnover of these factors is usually transcription dependent.^35, 36^ In the present study, we have demonstrated that Ets-1 is also controlled in this manner (i.e., its degradation induced by severe hypoxia is transcription dependent). We first observed that the decrease in Ets-1 protein level and increase in its transactivation activity happened simultaneously when the oxygen concentration was below 10%. We then observed that only transcriptionally inactive mutants of Ets-1 (i.e., the mutants that had lost the TAD domain and/or ETS domain) were resistant to severe hypoxia-induced degradation. The link between a transcription factor's enhanced degradation to its higher transactivation activity is not yet fully understood, but the ubiquitin-proteasome system always takes part in this process.^37, 38, 39^ In some cases, ubiquitination serves as a dual signal for the activation and the degradation of transcription factors; that is, while ubiquitination of these factors is required for their transactivation activity, it simultaneously promotes their degradation.^40, 41, 42^ Some other studies reveal that the signal-induced phosphorylation of transcription factors modulates their transactivation activity and ubiquitin-proteasome mediated degradation.^37, 38, 43, 44^ In many cases, these phosphorylation sites are located in the overlap between the TADs and the degron sequences.^44, 45^ The resulting phosphoamino acids promote gene transcription and are then recognized by E3 ubiquitin ligase, causing transcription factors to be degraded by the proteasomes. Our research showed that the ubiquitination and the transactivation activity of Ets-1 were both enhanced following hypoxia exposure. However, we did not determine whether inhibition of ubiquitination could block hypoxia-induced activation of Ets-1 or if hypoxia could induce a site-specific phophorylation leading to the activation and degradation of Ets-1. It is interesting that the regulatory sequence responsible for the activation and degradation of Ets-1 during hypoxia can be located in the TAD domain, for deletion of this domain blocked both processes. Further study is required to better understand how the enhanced degradation of Ets-1 during hypoxia is coupled with its elevated activity. An important component of our research will be to map the potential phosphorylation sites and ubiquitination sites regulated by hypoxia signal.

According to the research by Nishida *et al.*,^46^ protein inhibitor of activated STAT Y (PIASy) may serve as a link between the transactivation activity and the protein stability of Ets-1. They report that although PIASy prevents Ets-1 protein from ubiquitin-dependent proteasomal degradation,^46^ it also represses its transactivation activity.^47^ In agreement with our results, they identified the TAD domain and the C-terminal region, which contains the Ets domain, as requirements for Ets-1 protein degradation;^46^ but unlike our study, which focused on the effects of hypoxia, they investigated Ets-1 protein stability under normal culture conditions. Our findings show that the protein instability and the transactivation activity of Ets-1 are further elevated upon stimulation by severe hypoxia. Whether PIASy is responsible for transcription-dependent degradation of Ets-1 induced by hypoxia needs to be further explored.

A hypoxia-induced decrease in Ets-1 protein level has never been reported before, and we have not observed the decrease in many other cell lines (data not shown), including pancreatic *α*-cell line—*α*-TC6. This decrease seems to be specific to pancreatic *β*-cells. Research by Zhang *et al.*^48^ shows that Ets-1 inhibits glucose-stimulated insulin secretion in INS-1 cells and rat primary cultured islets. Similar results have been obtained in our laboratory (submitted for publication) and suggest that the degradation of Ets-1 may be an evolutionary mechanism preventing its over-activation and subsequent *β*-cell dysfunction. According to our research, *β*-cell dysfunction caused by constitutive high expression of Ets-1 can be reversed by knocking it down (submitted for publication). Therefore, the transient impairment of *β*-cell function would not be a barrier to the application of Ets-1 in islet graft protection.

Hypoxia induces cell apoptosis via the mitochondrial apoptotic pathway,^49, 50^ in which the disruption of the mitochondrial membrane potential is considered to be the initial event.^51, 52, 53^ In the present study, we have demonstrated that Ets-1 overexpression partially reversed hypoxia-induced mitochondrial membrane potential disruption. The way in which Ets-1 participates in apoptosis regulation is supported by its quick mobilization following hypoxia exposure. It is likely that Ets-1 regulates a diverse set of genes during hypoxia judging from its early responsive character, and a gene microarray is necessary to make a global gene expression analysis and to gain insight into its role in anti-apoptotic defense.

In conclusion, we have identified Ets-1 as an early response gene that regulates hypoxia-induced apoptosis in pancreatic *β*-cells. The relative insufficiency of active Ets-1 caused by its transcription-dependent degradation contributes to the susceptibility of *β*-cells to hypoxic injury. Controlled overexpression of Ets-1 in pancreatic *β*-cells during the immediate post-transplantation period may be a useful strategy for islet graft protection.

## Materials and Methods

### Reagents and antibodies

Actinomycin D, carbobenzoxy-Leu-Leu-leucinal (MG132), *β*-mercaptoethanol and mouse anti-*β*-tubulin antibody were purchased from Sigma-Aldrich (St. Louis, MO, USA). Rabbit anti-Ets-1, mouse anti-GFP and rabbit anti-ubiquitin antibodies were purchased from Santa Cruz Biotechnology (Santa Cruz, CA, USA). Rabbit anti-caspase-3, rabbit anti-cleaved caspase-3 and horseradish peroxidase-conjugated anti-mouse or rabbit secondary antibodies were purchased from Cell Signaling Technology (Danvers, MA, USA). Rabbit anti-HIF-1*α* antibody was purchased from Novus Biologicals (Cambridge, UK). Anti-rabbit light chain secondary antibody was purchased from Chemicon (Temecula, CA, USA).

### Plasmid constructions

The mouse Ets-1 expression plasmids pCMV5-Ets-1^WT^ and pEGFP-Ets-1^WT^ were constructed by inserting the full-length coding region of Ets-1 (transcript variant 1) into the pCMV5 vector (at Bgl II/SalI sites) and the pEGFP vector (at Bg lII/KpnI sites), respectively. pEGFP-Ets-1^1–138^, pEGFP-Ets-1^55–440^, pEGFP-Ets-1^136–440^, pEGFP-Ets-1^301–440^ and pEGFP-Ets-1^ΔETS^ were constructed by inserting the truncated sequences of the Ets-1 coding region into the pEGFP vector at Bgl II/KpnI sites. These truncated sequences were generated by PCR using pCMV5-Ets-1^WT^ as the template. The ΔPNT (pointed) and ΔTAD deletion mutations of the Ets-1 coding sequence were generated by overlap extension PCR (SOE PCR)^54, 55^ using pCMV5-Ets-1^WT^ as the template, and they were inserted into the pEGFP vector at Bgl II/KpnI sites to generate pEGFP-Ets-1^ΔPNT^ and pEGFP-Ets-1^ΔTAD^. The pEGFP-Ets-1^ΔExon VIII^ plasmid was constructed by inserting the full-length coding region of Ets-1 (transcript variant 2) into the pEGFP vector at Bgl II/KpnI sites.

To generate the VEGFR3 luciferase reporter construction VEGFR3-Luc, a 814-bp sequence within the 5′-regulatory region of the VEGFR3 gene that harbors Ets-1-binding motifs^25^ was amplified by PCR from mouse genomic DNA and was inserted into the pGL3-Basic vector (Promega, Madison, WI, USA) at KpnI/XhoI sites.

All constructions used in this study were sequenced and confirmed to be correct. The primer sequences used for cloning are presented in Supplementary Table 1.

### Luciferase reporter assay

To assess the transactivation activity of Ets-1, MIN6 cells were co-transfected with VEGFR3-Luc, pEGFP/pEGFP-Ets-1^WT^/pEGFP-ETS-1^ΔTAD^/pEGFP-ETS-1^ΔETS^ and a *β*-galactosidase expressing plasmid driven by the CMV promoter (Clontech Laboratories, Palo Alto, CA, USA).^56^ At 24 h following transfection, cells were maintained in normoxic condition or exposed to 2% O_2_ for 1 h, immediately washed with ice-cold PBS, and then lysed with Reporter lysis buffer (Promega). Cell debris was removed by centrifugation (12 000 g at 4 °C for 20 min) and the whole-cell lysate was then subjected to luciferase reporter assay.

Luciferase activity was measured with a luminometer (TD-20/20; Turner Designs, Sunnyvale, CA, USA) using a luciferase assay system (Promega). The Firefly luciferase activity was normalized with the *β*-galactosidase activity. Each experiment was performed in triplicate and repeated three times.

### Cell culture, gene transfer and hypoxia treatment

The mouse pancreatic *β*-cell line MIN6 (passage 16–30) was cultured in Dulbecco's modified Eagles medium (Invitrogen, Carlsbad, CA, USA) containing 25 mM glucose and supplemented with 15% fetal bovine serum (Invitrogen). The rat pancreatic *β*-cell line INS-1 (passage 60–80) was cultured in PRIM 1640 medium (Invitrogen) containing 11.1 mM glucose and supplemented with 10% fetal bovine serum. Both media were supplemented with 100 *μ*g/ml streptomycin, 100 U/ml penicillin and 50 *μ*mol/l *β*-mercaptoethanol. The pancreatic *α*-cell line—*α*-TC6—was cultured in Dulbecco's modified Eagle's medium supplemented with 10% fetal bovine serum, 100 *μ*g/ml streptomycin and 100 U/ml penicillin. Cells were maintained at 37 °C in a humidified incubator under 5% CO_2_/95% air.

For gene transfer, MIN6 cells were transiently transfected with plasmids using Lipofectamine 2000 (Invitrogen) according to the manufacturer's protocol, and INS-1 cells were infected with AdV (Adenovirus) -GFP or AdV-Ets-1 adenovirus (MOI=5), followed by further treatment.

For hypoxia treatments, cells or islets were transferred to a humidified incubator (Heracell 150i CO_2_ Incubator, Thermo Scientific, Waltham, MA, USA) supplied with the desired gas mixture (1–10% oxygen/94-85% N2/5% CO_2_).

### Pancreatic islets isolation

All animal studies were performed according to guidelines established by the Research Animal Care Committee of Nanjing Medical University. Animals used for islet isolation (8-week-old C57BL/6 mice and Sprague–Dawley rats) were purchased from the National Resource Center for Mutant Mice Model Animal Research Center of Nanjing University. Islets were isolated and cultured as described previously.^57^ At 6 h following isolation, islets were maintained under normoxic conditions or were subjected to hypoxic conditions. Total RNA and protein were then extracted after 1–4 h of hypoxia.

### Flow cytometry analysis of apoptosis and mitochondrial membrane potential (Δ*ψ*m)

Apoptosis was analyzed by annexin V/PI staining. The mitochondrial membrane potential was analyzed by JC-1 staining. After exposure to 2% O_2_, MIN6 cells were immediately washed with ice-cold PBS, collected and stained with annexin V-FITC/PI (annexin V-FITC apoptosis detection kit I, BD Biosciences, San Diego, CA, USA) and JC-1 (MitoProbe JC-1 Assay Kit, Life Technologies, Carlsbad, CA, USA) according to the manufacturers' protocols. A total of 2 × 10^4^ cells in each sample were analyzed using a FACSCalibur flow cytometer and Cellquest Pro software (Becton Dickinson Immunocytometry Systems, San Jose, CA, USA).

### RNA extraction, reverse transcription and qRT-PCR

Total RNA was extracted using TRIzol reagent (Invitrogen) according to the manufacturer's directions. Reverse transcription using ReverTra Ace-*α*-reagent (TOYOBO, Osaka, Japan) was performed to quantify relative amounts of mRNA using Oligo (dT) 20 primers. The SYBR Green Realtime PCR Master Mix (TOYOBO) and Light Cycler 480 II Sequence Detection System (Roche, Basel, Switzerland) were used for qRT-PCR. mRNA levels were normalized to *β*-actin. The sequences of the primers used in qRT-PCR are presented in Supplementary Table 2.

### Western blotting

After hypoxia exposure, cells or islets were immediately washed with ice-cold PBS and lysed with buffer containing 50 mM Tris-HCl (pH 8.0), 150 mM NaCl, 0.02% sodium azide, 0.1% SDS, 1 *μ*g/ml aprotinin, 1% NP-40, 1% deoxycholic acid sodium salt and 100 *μ*g/ml PMSF. Cell debris was removed by centrifugation (12 000 g at 4 °C for 20 min). The protein concentration was determined and samples of the protein were separated by SDS-PAGE, transferred to Immun-Blot PVDF membranes (Bio-Rad, Hercules, CA, USA), and incubated at 4 °C overnight with primary antibodies. The membranes were then incubated at room temperature with horseradish peroxidase-conjugated anti-mouse or anti-rabbit secondary antibodies for 1 h and analyzed using the ECL method.

### Immunoprecipitation

The MIN6 cells were transfected with pCMV5 or pCMV5-Ets-1. Twenty-four hours after transfection, the cells were exposed to 2% O_2_ for 1 h or 2 h, immediately washed with ice-cold PBS and then lysed with RIPA buffer containing 20 mM Tris-HCl (pH 7.5), 150 mM NaCl, 1 mM EDTA, 1 mM EGTA, 1% Triton X-100, 2.5 mM sodium pyrophosphate, 1 mM *β*-glycerolphosphate, 1 mM sodium orthovanadate, 100 *μ*g/ml PMSF and a complete protease inhibitor (Roche Molecular Biochemicals, Indianapolis, IN, USA). Cell debris was removed by centrifugation (12 000 g at 4 °C for 20 min). The lysates were pre-cleared with protein A/G Plus agarose beads and then incubated with anti-Ets-1 antibody and protein A/G Plus agarose beads for 9 h. The precipitates were separated from the beads by heating in 1 × sample buffer in a boiling water bath for 5 min. The extracted proteins were then analyzed by western blotting. The anti-rabbit light chain secondary antibody was used following incubating with anti-Ets-1 primary antibody for immunoblotting of Ets-1 protein.

### Statistical analysis

Comparisons were performed using Student's *t*-test between pairs of groups. Results are presented as means±S.D. *P*<0.05 was considered to be statistically significant.

## Abbreviations

AdV: Adenovirus
CMV: Cytomegalovirus
Ets-1: v-ets erythroblastosis virus E26 oncogene homolog 1
HIF-1: Hypoxia-inducible factor 1
MG132: Carbobenzoxy-Leu-Leu-leucinal
PI: Propidium Iodide
PNT: Pointed
qRT-PCR: quantitative real-time polymerase chain reaction
TAD: transactivation domain
VEGFR: Vascular endothelial growth factor receptor
