# Supplementary material for: Ets-1 as an early response gene against hypoxia-induced apoptosis in pancreatic β-cells
Source: Cell Death Dis. 2015 Feb 19;6(2):e1650–. doi: 10.1038/cddis.2015.8 (PMC4669796; doi:10.1038/cddis.2015.8)
Supplement: Supplementary Figure Legend [file cddis20158x1.doc]

**Supplementary Figure Legend**

**Supplementary Figure 1**

*In vitro* assessment of apoptosis in Min6 cells exposed to severe hypoxia. MIN6 cells were exposed to 2% O_2_ for 12, 16 and 24 h, respectively. **(a)** The cells were exposed to hypoxia and then stained with Annexin Ⅴ-FITC/PI and analyzed by Flow cytometry to determine the percentage of apoptotic cells. Dots in the lower right quadrant indicate Annexin Ⅴ^+^/PI^-^, early apoptotic cells, while dots in the upper right quadrant indicate Annexin Ⅴ^+^/PI^+^, late apoptotic cells. **(b)** Statistical graph of apoptotic cells as percentages. The Y axis, (i.e., % apoptotic cells) indicates the percentage of early plus late apoptotic cells. The average values and standard deviations (n=3) are shown. ** indicates P < 0.01 compared with the normoxic group. **(c)** Total proteins from cells exposed to hypoxia were extracted and analyzed by western blotting using the indicated antibodies. The upper panel shows a representative western blot. The lower panel shows the relative quantification of normalized Cleaved Caspase-3 and Caspase-3 levels to β-tubulin. The average values and standard deviations (n=3) are shown. * and ** indicate P < 0.05 and P < 0.01, respectively, compared with the normoxic group.
