# Supplementary material for: Ets-1 as an early response gene against hypoxia-induced apoptosis in pancreatic β-cells
Source: Cell Death Dis. 2015 Feb 19;6(2):e1650–. doi: 10.1038/cddis.2015.8 (PMC4669796; doi:10.1038/cddis.2015.8)
Supplement: Supplementary Table 1 [file cddis20158x2.doc]

**Supplementary Table 1. Primers for cloning**

| **NAME** | **SEQUENCES (5’-3’)** | |
| --- | --- | --- |
|  | **Forward** | **Reverse** |
| pCMV5-Ets-1^WT^ | GAAGATCTATGAAGGCGGCCGTCGATC | ACGCGTCGACCTAGTCAGCATCCGGCT |
| pEGFP-Ets-1^WT^ | GAAGATCTATGAAGGCGGCCGTCGATC | GGGGTACCGTCAGCATCCGGCTTTACAT |
| pEGFP-Ets-1^1-138^ | GAAGATCTATGAAGGCGGCCGTCGATCT | CGGGGTACCTTTCACATCCTCTTTCTGCAG |
| pEGFP-Ets-1^55-440^ | GAAGATCTGGTTTCACAAAAGAACAGCAG | GGGGTACCGTCAGCATCCGGCTTTACAT |
| pEGFP-Ets-1^136-440^ | GAAGATCTGATGTGAAACCATATCAGGTT | GGGGTACCGTCAGCATCCGGCTTTACAT |
| pEGFP-Ets-1^301-440^ | GAAGATCTAAGGGCACCTTCAAGGACTAT | GGGGTACCGTCAGCATCCGGCTTTACAT |
| pEGFP-Ets-1^ΔETS^ | GAAGATCTATGAAGGCGGCCGTCGATCT | CGGGGTACCGGGCTTGTGGTTGGGCAGG |
| pEGFP-Ets-1^ΔExon Ⅶ^ | GAAGATCTATGAAGGCGGCCGTCGATCT | CGGGGTACCGTCAGCATCCGGCTTTACAT |
| pEGFP-Ets-1^ΔPNT^ | F1: GAAGATCTATGAAGGCGGCCGTCGATCTCAAGC  F2: GCTACTTTCAGTGATGTGAAACCATATCAG | R1: TATGGTTTCACATCACTGAAAGTAGCTTTC  R2: CGGGGTACCCTAGTCAGCATCCGGCTTTAC |
| pEGFP-Ets-1^ΔTAD^ | F1: GAAGATCTATGAAGGCGGCCGTCGATCTCAAGC  F2: GGTAAACTCGGGGGCCAGGACTCTT | R1: CTGGCCCCCGAGTTTACCCTCTTTCTGCAGGATC  R2: CGGGGTACCCTAGTCAGCATCCGGCTTTAC |
| VEGFR3-Luc | CGGGGTACCCATTCCTGTTCTGTGGTTGAG | CCGCTCGAGGCGGCCGCAGCTCTAGGCT |
