# Supplementary material for: Ets-1 as an early response gene against hypoxia-induced apoptosis in pancreatic β-cells
Source: Cell Death Dis. 2015 Feb 19;6(2):e1650–. doi: 10.1038/cddis.2015.8 (PMC4669796; doi:10.1038/cddis.2015.8)
Supplement: Supplementary Table 2 [file cddis20158x3.doc]

**Supplementary Table 2. Primers for qRT-PCR**

| **NAME** | **SEQUENCES (5’-3’)** | |
| --- | --- | --- |
|  | **Forward** | **Reverse** |
| Ets-1 (Mouse) | AATCGTCGGCATCATAGCA | TGTTTCGGGTAGCGGTTTA |
| Ets-1 (Rat) | AGAAAGAGGATGTGAAACC | CATGCTCGATACCATAG |
| VEGFR1 | CAACGTCCAACAGGATGGGA | TGGAGTTCGGTGAAAGCTCC |
| VEGFR2 | GAATGTCCCACCCCAGATCG | GCTTGGATGACCAGCGTACT |
| VEGFR3 | AGACCCCGACTATGTCCGAA | GTATGGAGAGGCCCCCAATG |
| β-actin (mouse) | AGGCCAACCGTGAAAAGATG | AGAGCATAGCCCTCGTAGATGG |
| β-actin (Rat) | AGCCATGTACGTAGCCATCC | CTCTCAGCTGTGGTGGTGAA |
