# Supplementary figures and images for: Ets-1 as an early response gene against hypoxia-induced apoptosis in pancreatic β-cells
Source: Cell Death Dis. 2015 Feb 19;6(2):e1650–. doi: 10.1038/cddis.2015.8 (PMC4669796; doi:10.1038/cddis.2015.8)

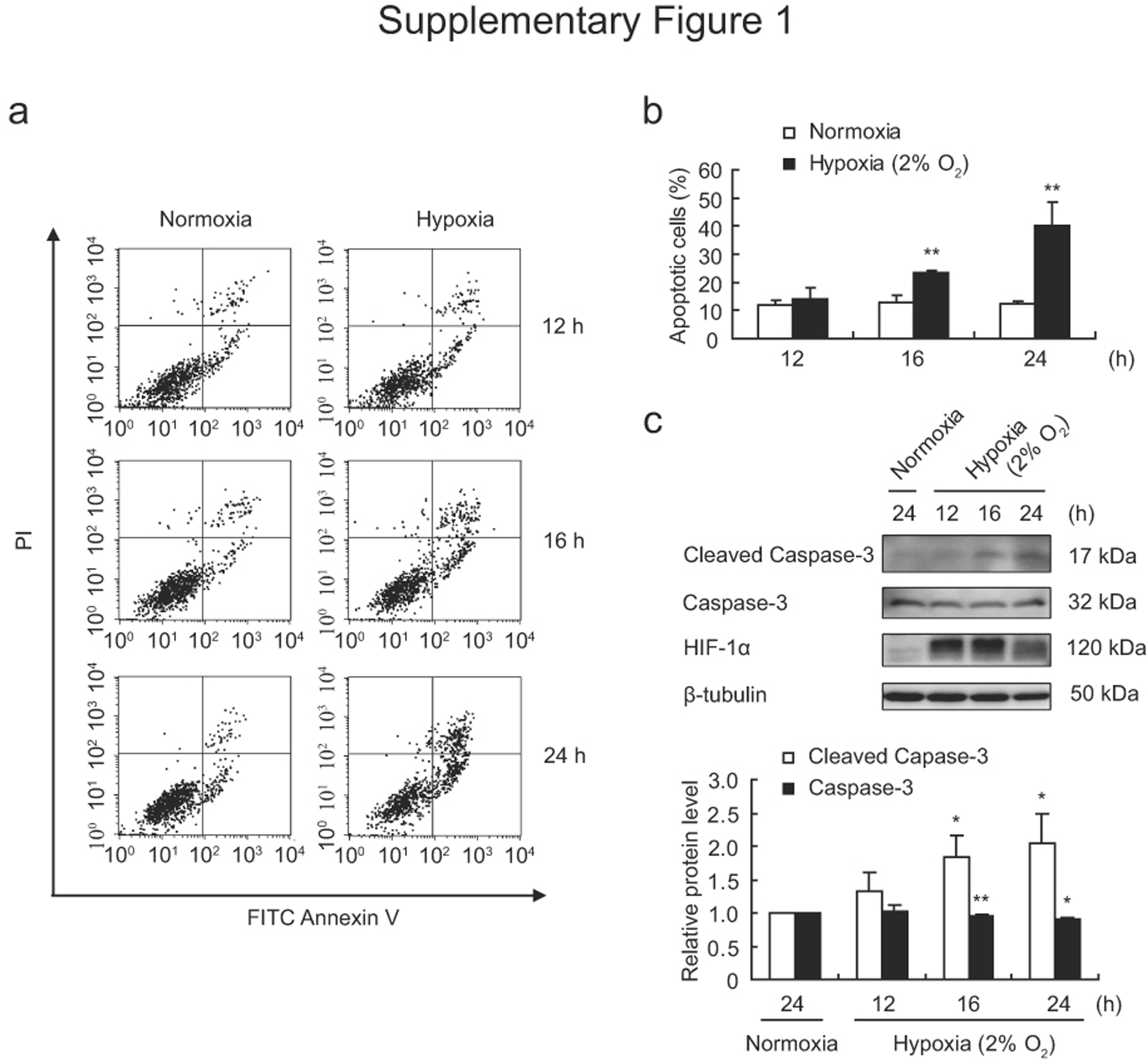

Supplement: Supplementary Figure 1 [file cddis20158x4.tif]
